# Vitamin B12 deficit and development of geriatric syndromes

**Published:** 2013-03-30

**Authors:** José Mauricio Ocampo Chaparro

**Affiliations:** Department of Family Medicine, Faculty of Health, Universidad del Valle, Cali, E-mail: jmocampo2000@yahoo.com.ar

**Keywords:** Elderly, vitamin B12 deficiency, Geriatric syndromes

## Abstract

Vitamin B12 deficiency or cyanocobalamin is a common condition in the elderly. It is repeatedly overlooked due to multiple clinical manifestations that can affect the blood, neurological, gastrointestinal, and cardiovascular systems, skin and mucous membranes. The various presentations of vitamin B12 deficiency are related to the development of geriatric syndromes like frailty, falls, cognitive impairment, and geriatric nutritional syndromes like protein-energy malnutrition and failure to thrive, in addition to enhancing aging anorexia and cachexia. Therefore, interventions must be developed to include their screening and diagnosis to make early and appropriate treatment to prevent its complications before they become irreversible.

## Abstract (es)

El déficit de vitamina B12 o cianocobalamina es una condición frecuente en adultos mayores que reiterativamente se pasa por alto debido a múltiples manifestaciones clínicas que pueden afectar el sistema hematológico, neurológico, gastrointestinal, cardiovascular, piel y mucosas. Las diversas presentaciones del déficit de vitamina B12 se encuentran relacionadas con el desarrollo de síndromes geriátricos como fragilidad, caídas, deterioro cognoscitivo, síndromes nutricionales geriátricos como desnutrición proteicocalórica y falla para prosperar, además de potenciar la anorexia del envejecimiento y caquexia. Por consiguiente, se deben desarrollar intervenciones que incluyan su tamizaje y diagnóstico con el objetivo de realizar un tratamiento temprano y oportuno para evitar que sus complicaciones se conviertan en irreversibles.

## Introduction

Vitamin B12 deficit or cyanocobalamin (Cbl) has a great variety of clinical manifestations reflected in the compromise of different organic systems like the hematological, neurological, gastrointestinal, skin, mucosa, and cardiovascular systems^1^. These manifestations are in turn related in the genesis of different geriatric syndromes like frailty, falls, cognitive decline, and geriatric nutritional syndromes like failure to thrive and anorexia of aging^2^.

## Case report

Herein, we report a case of a 71-year-old female patient hospitalized for anemia, motor incoordination, gait disturbance, weakness, and decline of her functional state. She was asymptomatic eight months prior to the day she was hospitalized, when she had asthenia, adynamia, hyporexia, symptoms of dizziness, and vertigo. Six months prior to admission, the symptoms increased, with onset of loss of balance, and postural instability. The patient also developed deterioration for walking, with repeated falls, which led her to stop walking for fear of falling, and limited her physical daily living activities to simply transferring from the chair to the bed. Likewise, it was noted that upon flexing her neck, she reported feeling an electrical discharge irradiating from her back to her legs. Two months before hospital admission, she noted lesions on the tongue, which produced a burning sensation and pain when swallowing foods - leading her to decreased intake of food and a loss of 5 kg coupled with increased feeling of weakness in her general state. She consulted with her local hospital, where she was diagnosed with paraparesis and anemia to continue studies.

As personal antecedents, she revealed hypertension managed with captopril 50 mg every 12 h. She did not report alcohol consumption, vegetarian nutritional habits, or other personal or family antecedents of importance.

**Figure 1:**
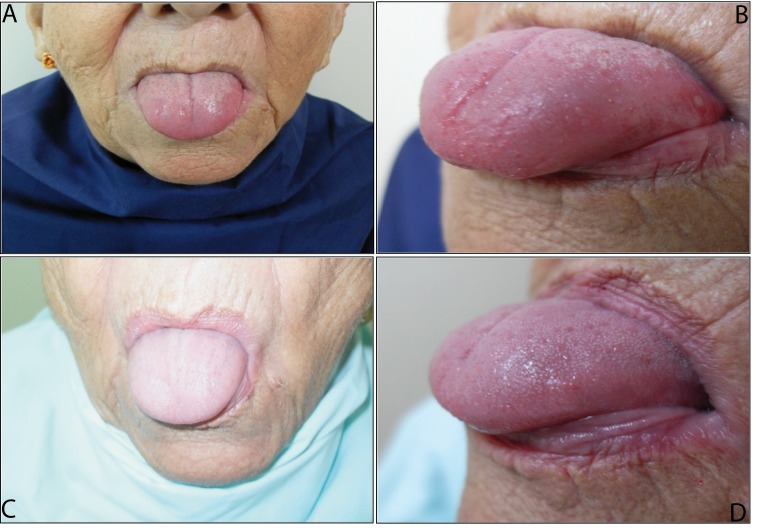
Glossitis due to vitamin B12 deficiency, before (A, B) and after treatment (C, D)

Upon physical exam for admission, she was in poor general state, pale, and marked loss of muscle mass. Blood pressure was 130/80 mm/Hg, without orthostasis, respiratory rate 17 per min and heart rate 85 per min. Her weight before developing the diseasewas 70 kg; her current weight is 60 kg, height 1.65 m. The oral cavity showed smooth, shiny, reddish depapillated tongue with ulceration at lateral level (Fig. 1 A and B).

Neurological exam revealed that she was alert and oriented in all three spheres, no cranial nerve involvement, or abnormal movements. She had muscle weakness grade 3/5 and spasticity in all four limbs, tone and tropism diminished. The superficial sensitivity, touch, pain, and temperature systems were normal. Deep sensitivity was altered, with lack of vibratory sensation of the sense of position in upper and lower limbs, numbness in hands and feet, and positive Lhermitte's sign. Gait was characteristic of sensory ataxia with postural instability, broad support polygon, positive Romberg's sign, diminished osteotendinous reflexes especially in lower limbs and bilateral flexor plantar reflex present. Different para clinical studies were carried out (Table 1). Urinalysis, serology, and direct Coombs test were also performed under normality parameters. Additionally, extended blood test was conducted, revealing red cell anisocytosis, macrocytosis, and poikilocytosis. White series with hypersegmented neutrophils and platelet series without alterations were found. Upper gastrointestinal endoscopy was performed with the biopsy showing chronic atrophic gastritis positive for *Helicobacter pylori.*

**Table 1:**
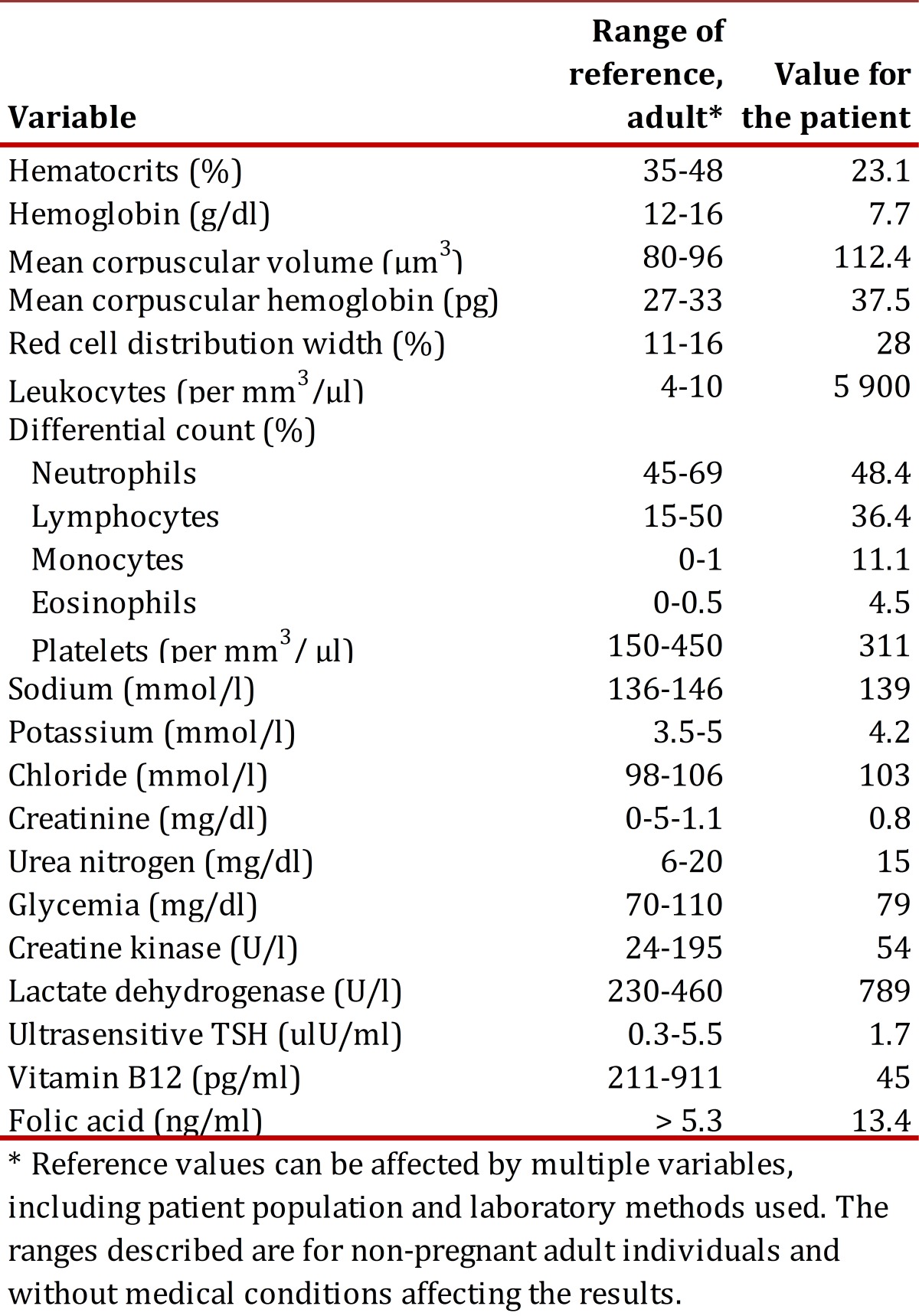
Laboratory results * Reference values can be affected by multiple variables, including patient population and laboratory methods used. The ranges described are for non-pregnant adult individuals and without medical conditions affecting the results.

Geriatric assessment scales were applied, showing: mini-mental exam (28/30), geriatric depression scale (4/15), physical aspect of Barthel's scale of activities of daily living (60/100), JH Downton's scale of risk of falling in the elderly (3 points), and assessment of nutritional state through the mini nutritional assessment (16/30 points).

**Figure 2:**
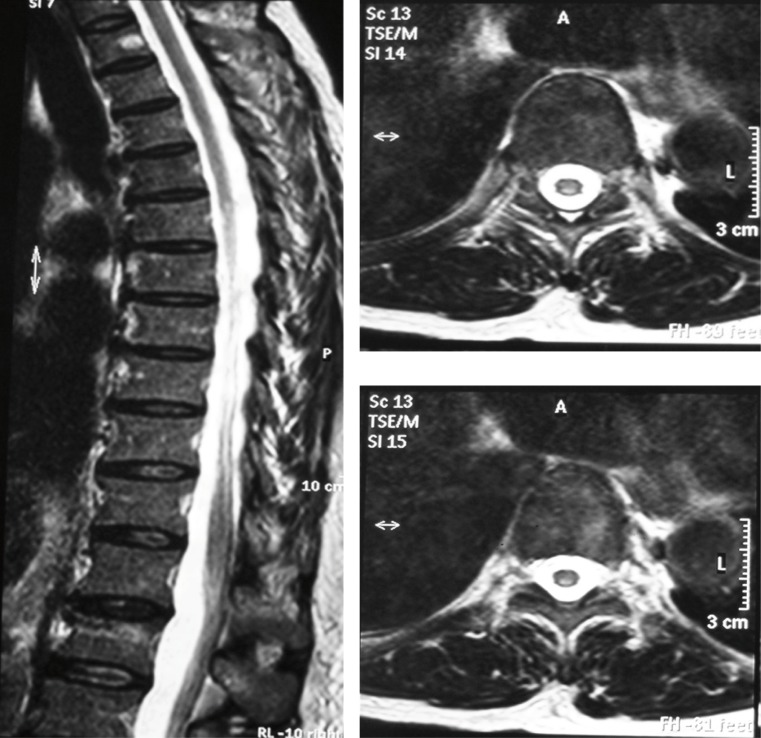
Cervical and thoracic spine, NMR image in T2 sequence. (A) Sagittal plane, shows hyperintensity in topography of posterior spinal cords from C1 to T11. (B, C) Axial plane at C1 and T8 levels, respectively, evidences hyperintensity symmetrically compromising lateral and posterior spinal cords.

Vitamin B12 deficit diagnosis was performed with hematological manifestations given by macrocytic anemia, neurological manifestations by ataxic gait, and in skin and mucosa by Hunter's glossitis. Because of the neurological alterations in association with ataxic gait and low levels of vitamin B12, nuclear magnetic resonance of the cervical and thoracic spine was requested, which documented images compatible with sub-acute combined degeneration of the spine (Fig. 2).

Replacement with Cbl was begun, showing after three weeks increased Hemoglobin values with decreased levels of lactate dehydrogenase indicating improvement in ineffective erythropoiesis. Glossitis was resolved after a month of establishing Cbl reposition Fig. 1 (C and D). After three months of treatment, gait improved with the patient walking independently; sensitive symptomatology also improved.

## Discussion

The first description of vitamin B12 deficit was made by British physician Thomas Addison in 1855, who called it pernicious anemia and it was described as a disease that manifested itself with macrocytic anemia, glossitis, and neurological symptoms similar to the case presented^3^.

Vitamin B12 is part of a group of hydro-soluble vitamins, an essential nutrient that must be obtained in the diet. This vitamin exerts its physiological action through two enzymatic pathways: the first acts as a co-factor for the methionine synthase enzyme that converts homocysteine into methionine and the second acts upon L-methylmalonyl Coenzyme A (CoA) mutase enzyme to convert methylmalonyl-CoA into succinyl-CoA4. Thus, B12 deficit can lead to increased homocysteine, which has vasculotoxic, neurotoxic, and carcinogenic action, and of the methylmalonic acid with neurotoxic effects that affect myelin sheaths and axons^4^.

Also, a typical diet in a western nation offers between 5 and 15 µg of vitamin B12 daily. The recommended nutritional intake of vitamin B12 is from 2 to 3 µg/day and the body reserves are from 2 to 5 mg. For this reason, between 2 and 5 years are required from the onset of the B12 deficit until the first clinical manifestations are noted, which is explained by its important storage at hepatic level (>1.5 mg) and by its enterohepatic cycle^5^.

The B12 deficit can affect any age group, although it is most frequent in the elderly because of the high prevalence of atrophic gastritis, which is caused by autoimmune mediation, as well as the concomitant presence of *H. pylori* infection^6^. In addition, atrophic gastritis leads to destruction of gastric parietal cells, which produce hydrochloric acid and intrinsic factor; the latter is a necessary glycoprotein that permits Cbl absorption at the terminal ileum level. Both atrophic gastritis and the presence of *H. pylori* were found in the patient and they probably explain the etiology of the Cbl deficit.

Among the causes of B12 deficit in the elderly, the following are included: inadequate intake noted in vegetarian individuals, mal-absorption due to gastrointestinal alterations like atrophic gastritis that causes hypochlorhydria, antecedent of gastrectomy or ileal resection, bariatric surgery, Crohn's disease, and intestinal mal-absorption syndrome^4^.

The intestinal mal-absorption syndrome bears special importance, given that it is explained among 60 and 70% of the cases of vitamin B12 deficit in the elderly^7^. It is produced by bacteria over-growth, which is favored by frequent achlorhydria in the elderly, decreased intestinal motility, exocrine pancreatic insufficiency, and intake of antacids, which cause diminished capacity to liberate vitamin B12 bonded to foods or proteins.Other causes of B12 deficit are autoimmune diseases like diabetes mellitus and thyroid diseases, intake of medications - especially proton pump inhibitors, phenytoin, biguanides, and aspirin, along with exposure to anesthetic gases like nitrous oxide and chronic consumption of alcohol^1^.

Cyanocobalamin deficiency can have complex clinical manifestations due to compromise in multiple organic systems and can be associated to the development of different geriatric syndromes (Table 2).

**Table 2:**
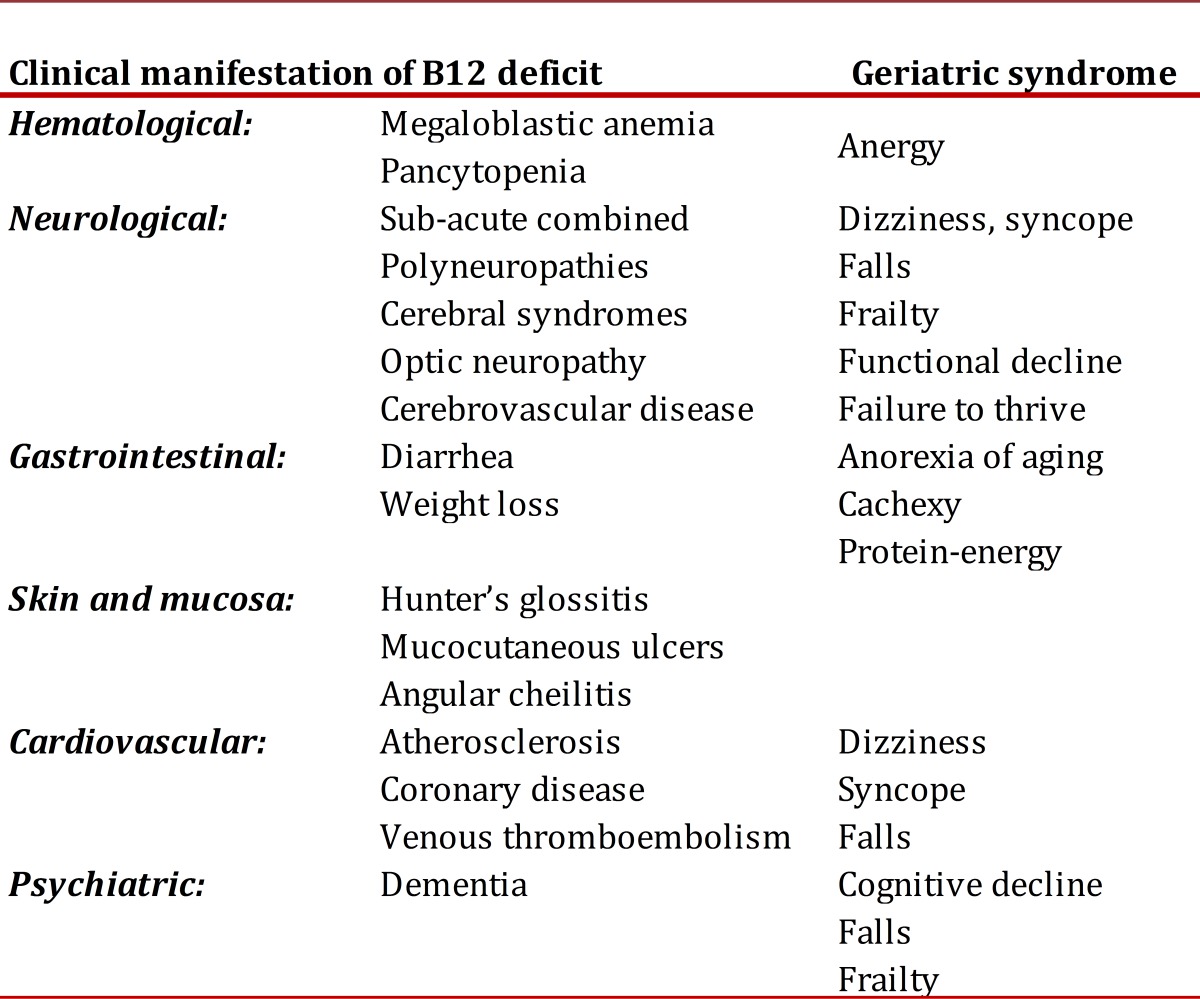
Relationship between clinical manifestation of B12 deficit and geriatric syndromes

Within the hematologic symptoms, patients may debut with pancytopenia, although some may not develop anemia or macrocytosis; however, the most frequent presentation is macrocytic anemia as presented by the patient.

Regarding neurological symptoms, these vary according to the structure affected, including peripheral nerve, spinal cord, brain, and optic nerves, which develop during a several-month period^8^. The severity of the neurological manifestations is directly correlated to the duration of the symptoms and inversely correlated to Hemoglobin value.

Peripheral neuropathy is the most frequent neurological manifestation; it appears with paresthesias and numbing of the feet and legs, accompanied by hyporeflexia, alteration in superficial sensitivity with boot distribution and compromise of vibratory sensitivity; it is then developed similarly in the hands, along with distal weakness of lower limbs, as with the case presented^1^.

Myelopathy secondary to Cbl deficit, denominated sub-acute combined degeneration of the spine, was one of the cardinal manifestations of the case described along with macrocytic anemia. It is characterized by marrow involvement that affects the posterior and lateral spinothalamic tract, which is initially present at lower cervical and upper thoracic spine level and advances towards the cranial and caudal directions, as well as forward, invading lateral and anterior columns^8^.

Clinical manifestations are given by disorder in deep sensitivity at lower limb level with hypoesthesia, paresthesias, decreased proprioception, and instability in walking with broad support base due to sensory ataxia, which appear symmetrically^7^. In advanced stages tetraparesis or spastic paraparesis and contractures may develop^6^.

Morrow involvement due to Cbl deficit is associated to the presence of different geriatric syndromes like dizziness and syncope, both related to falls, fear of falling, frailty, and failure to thrive^2^. With respect to the case presented, the patient developed gait with characteristics of sensory ataxia, which caused in her a syndrome of falls and fear of falling, with the subsequent restriction in her activities of daily living, leading to frailty and − lastly − functional decline with severe dependence according to Barthel's scale.

When the cervical segments are affected, equivalent symptomatology is observed in the upper limbs, and characteristic presence of Lhermitte's sign, manifested by the patient. This sign is triggered by flexing of the neck, characterized by electrical sensation along the rachis, and it is considered an indicator of demyelization of the posterior spinal cords^7^.

The neuropathological findings in the sub-acute combined degeneration of the spine are given by degeneration of the myelin sheaths, with formation of intramyelinic vacuoles and separation of the myelin sheets. Spongiform demyelization is produced in scattered plates that start at the level of the lateral and posterior columns of the cervical and thoracic spinal cord, which can often affect the anterior columns with axonal degeneration and gliosis in advanced stages of the disease^8^.

The NMR images show focal and symmetric hyperintensity in the posterior half of the spinal cord visible in T2 sequence, explained by increased water content, as illustrated in (Fig. 2). Differential diagnoses for these lesions observed under magnetic resonance include infections (tabes dorsalis, human immunodeficiency virus, and herpes Zoster), neoplasms (lymphoma, paraneoplastic myelopathy), vascular (arterial or venous ischemia, arteriovenous malformation of spinal cord), post-radiation myelitis, multiple sclerosis, acute transverse myelitis, and syringomyelia^8^.

Regarding psychiatric symptoms, these have a broad presentation range from depression to dementia, which are part of the cognitive decline syndrome^2^. They appear as the consequence of multiple foci of demyelization at the level of the frontal and parietal white matter and corpus callosum.

The symptomatology is varied and may present mood disorders, compromise in executive function, and memory, confusion, agitation, visual and auditory hallucinations.

Nevertheless, although B12 deficit is frequently found in the elderly with dementia or depression, administration of Cbl has not shown improved cognitive function; consequently, it cannot be stated that said vitamin deficiency behaves as a causal factor^6^. In effect, even though the patient had Cbl deficit, the assessment scales do not evidence deterioration of their cognitive functions or depression.

Additionally, manifestations in mucosa include glossodynia, recurrent ulcers, dysgeusia, lingual paresthesia, stomatitis, and angular cheilitis. However, Hunter's glossitis is the most frequent presentation, found in up to 25% of the cases and it is characterized by a reddish, smooth tongue with atrophy of lingual papillae and alterations of taste; all the aforementioned was present in the patient^5^. Said abnormalities can lead the elderly to develop nutritional syndromes like protein-energy malnutrition and failure to thrive, as well as enhance anorexia of aging and cachexy^2^.

In the case presented, it was evident that alteration of the mucosa lead to unintended weight loss, an important characteristic because it is one of the main routes of ingress for the frailty syndrome and, likewise, for different geriatric nutritional syndromes to originate^5^. It is worth mentioning that the patient presented a state of malnutrition according to the mini nutritional assessment scale.

Finally, this case shows that the elderly group constitutes a vulnerable population to develop vitamin B12 deficit. Its high prevalence, as well as the insidious course of symptoms and multiple clinical manifestations, including the development of geriatric syndromes turns it into an important condition in public health. Thereby, interventions should be developed that include its screening and diagnosis, with the aim of conducting early and timely treatment to keep its complications from becoming irreversible.
